# The impact of surgical timing on outcome in acute appendicitis in adults: a retrospective observational population-based cohort study

**DOI:** 10.1097/JS9.0000000000001528

**Published:** 2024-05-03

**Authors:** Konstantin Uttinger, Philip Baum, Johannes Diers, Daniel Seehofer, Christoph-Thomas Germer, Armin Wiegering

**Affiliations:** a Department of General, Visceral, Transplant, Vascular and Pediatric Surgery at Würzburg University Medical Centre; b Department of Visceral, Transplant, Thoracic and Vascular Surgery, Leipzig University Medical Centre, Leipzig; c Department of Thoracic Surgery, Thoraxklinik at Heidelberg University Medical Centre, Heidelberg, Germany; d Marienkrankenhaus, Hamburg; e Comprehensive Cancer Centre Mainfranken, Würzburg University Medical Centre; f Department of Biochemistry and Molecular Biology, University of Würzburg, Würzburg

**Keywords:** acute appendicitis, clinical endpoint, complicated appendicitis, observational study, time from admission to surgery, timing of surgery

## Abstract

**Background:**

Acute appendicitis is a global disease with high incidence. The main objective was to assess the association between time from admission to surgery (TAS) and surgery during emergency hours with operative outcome in light of conflicting evidence.

**Methods:**

This is a retrospective population-wide analysis of hospital billing data (2010–2021) of all adult patient records of surgically treated cases of acute appendicitis in Germany by TAS. The primary outcome was a composite clinical endpoint (CCE; prolonged length of stay, surgical site infection, interventional draining after surgery, revision surgery, ICU admission and/or in-hospital mortality). Cases of complicated appendicitis were identified using diagnosis (ICD-10) and procedural codes (resection beyond appendectomy).

**Results:**

855 694 patient records were included, of which 27·6% (236,481) were complicated cases of acute appendicitis. 49·0% (418,821) were females and median age was 37 (interquartile range 22·5–51·5). Age, male sex, and comorbidity were associated with an increased proportion of CCE and in-hospital mortality. TAS was associated with a clinically relevant increase of CCE after 12 h in complicated appendicitis [Odd’s ratio (OR), 1·19, 95% CI: 1·14–1·21] and after 24 h in uncomplicated appendicitis (OR 1·10, 95% CI: 1·02–1·19). Beyond the primary endpoint, the proportion of complicated appendicitis increased after TAS of 72 h. Surgery during emergency hours (6 pm–6.59 am) was associated with an increase of CCE and mortality (OR between 1·14 and 1·49). Age, female sex, night-time admission, weekend admission, a known previous surgery, obesity, and therapeutic anticoagulation were associated with delayed performance of surgery.

**Conclusion:**

This work found an increase of a CCE after TAS of 12 h for complicated appendicitis and an increase of the CCE after TAS of 24 h for uncomplicated appendicitis with a stable proportion of complicated appendicitis in these time windows. Both CCE and mortality were increased if appendectomy was performed during emergency hours.

## Introduction

Highlights

- In complicated appendicitis, in-house delay >12 h led to an increased composite clinical endpoint.
- In uncomplicated appendicitis, in-house delay >24 h led to an increased composite clinical endpoint.
- Surgery during emergency hours was associated with inferior in-hospital outcome.
- Factors associated with delayed surgery included night-time and weekend admission.

Acute appendicitis is a widespread condition with incidence rates of up to 230 per 100 000 people, with increasing global incidence^1^. Traditionally, acute appendicitis has been regarded as a medical emergency assuming appendiceal obstruction, which leads to perforation over time. Consequently, emergency appendectomy in the form of laparoscopic appendectomy has been advocated as state of the art due to the observed high morbidity and mortality rates associated with complicated, especially perforated appendicitis^2^. Despite recent studies challenging the notion that uncomplicated appendicitis invariably progresses to a complicated form^3,4^, highlighting that these two forms might display two different entities^5^, appendectomy is still considered the gold standard for both uncomplicated and complicated cases, while for uncomplicated cases, increasing evidence supports the noninferiority of nonoperative management^3,4^.

In terms of urgency, current clinical guidelines^6^ were established based on existing evidence, which, while of differing quality, has suggested a time-dependent increase of complications or perforation rates in time windows of in-hospital delay between 6 and 48 h^7–17^, or has found no such association^18–22^.

In practice, not all hospitals have 24/7 surgical capabilities, leading to the need for triage between different patients and levels of urgency. Overall, there is scarce evidence of in-hospital delay with stratification between uncomplicated and complicated appendicitis.

Though night-time appendectomy has not been associated with higher complication rates^22–25^, overall emergency surgery has been reported to be associated with a higher risk of mortality^26^.

It was the objective of this analysis to identify time intervals of time from admission to the beginning of surgery in acute appendicitis, during which no clinically relevant increase of a predefined composite clinical endpoint (CCE) is observed. It was a predefined hypothesis that stratification between an uncomplicated form of appendicitis and a complicated form of appendicitis reveals differing time windows, during which no such increase is found.

For this comprehensive analysis of an association between in-hospital patient outcome and time from admission to surgery (TAS) as well as timing of surgery (emergency vs nonemergency hours), we conducted a nationwide analysis using billing data spanning 12 years (2010–2021) and including over 850 000 patients who underwent surgery for acute appendicitis. The primary endpoint was a CCE; involving a prolonged postoperative length of stay, the occurrence of surgical site infection, ICU admission, interventional draining after surgery, revision surgery, and/or in-hospital mortality, secondary endpoints were in-hospital mortality and the proportion of complicated appendicitis. CCE and in-hospital mortality were stratified by the intraoperative finding of uncomplicated and complicated appendicitis.

### Data acquisition and definition of the study cohort

This is a retrospective, nationwide cohort study of anonymized diagnosis-related groups billing data provided by the ‘Statistische Bundesamt’ (Federal Statistical Office in Germany), comprising all billing records from all hospitals in Germany, regardless of hospital status or financing structure. Data acquisition was conducted in close contact with the Research Centre of the Federal Statistical Office (Data source: Diagnosis-Related Group Statistics (2010–2021)) and in accordance with their guidelines for handling highly sensitive patient-record data. No ethical vote was necessary for this large-scale, nationwide cohort analysis, as defined by German data safety law^27^.

**Figure 1:**
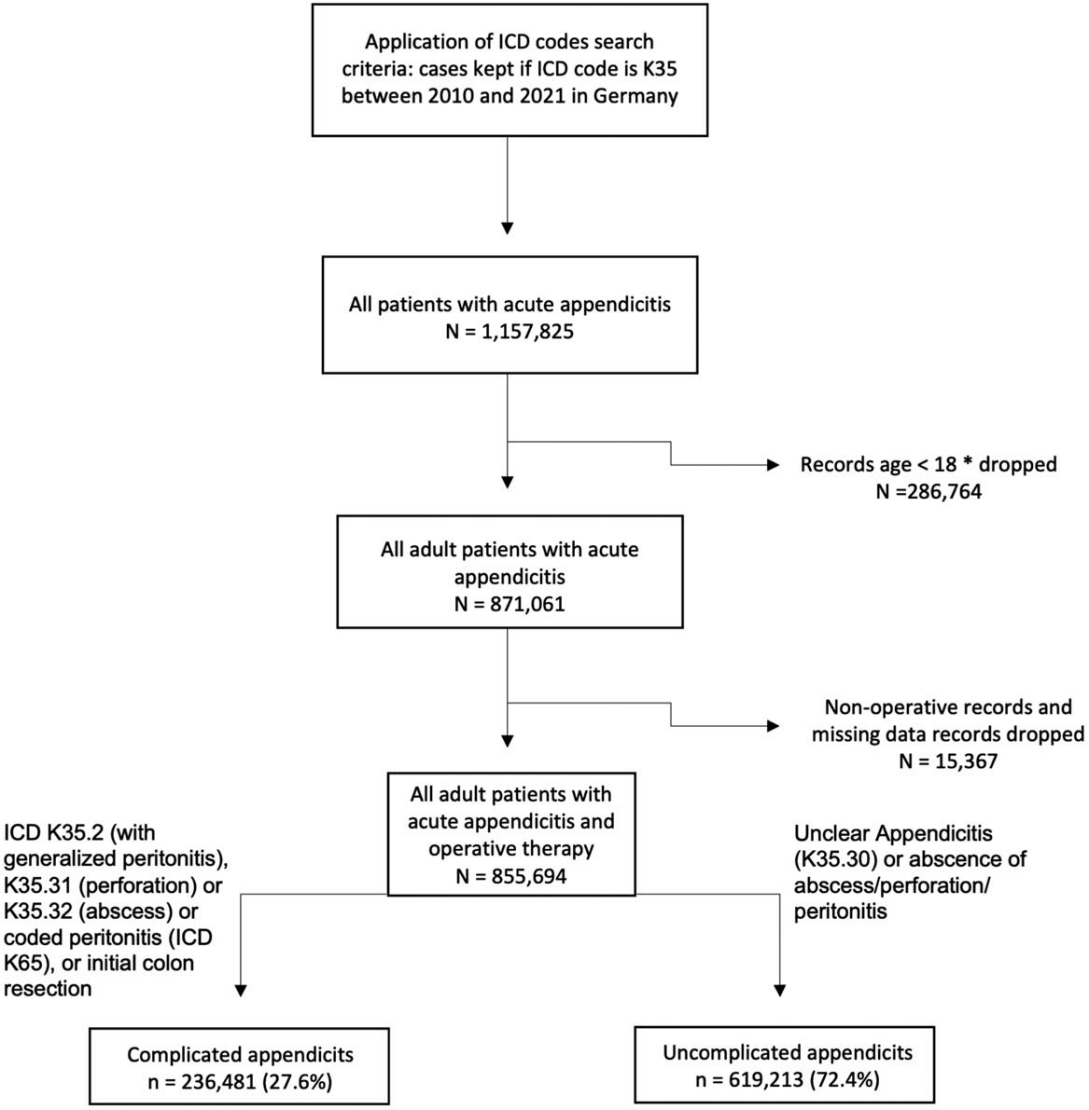
Flowchart of inclusion criteria and distinction of complicated vs. uncomplicated appendicitis. *or records with missing data; non-adult complete records mounted up to 276 816 admissions.

Identification of patients for this study was done using coding according to the International Classification of Diseases (ICD-10, German Modification, GM). All patients admitted for acute appendicitis (K35) were included (Supplementary Table 1, Supplemental Digital Content 1, http://links.lww.com/JS9/C472 and Fig. 1). Each patient record contained data on age, sex, procedural codes (‘Operationen- und Prozedurenschlüssel’, OPS), main and secondary diagnoses, length of stay, and reason for admission and discharge. Only complete data records were analyzed. All patients younger than 18 years at the time of admission were dropped and duplicates were identified, of which, in case of occurrence, one was chosen randomly for further analysis.

### Identification of surgical procedures and cases of complicated appendicitis

For each patient record, all OPS codes were accessible including associated time variables. If none of the OPS codes for any procedure/surgery (Supplementary Table 1, Supplemental Digital Content 1, http://links.lww.com/JS9/C472) was coded, the case was dropped for further analysis. Time of admission to the hospital was coded with exact time and date. Time variables reflect the beginning of the OPS procedure as specified in the billing data process. It is not verifiable if this, in case of surgeries, is the time of anesthesiologist preparations or the actual start of surgery, since individual hospitals code this differently. Time from admission to surgery (TAS) was defined as time from admission to the appendectomy procedure, which was independent of the extent of the resection.

Complicated appendicitis was defined either as ICD K35.2, K35.31, K35.32, or as unknown extent of K35 (acute appendicitis) with additional coding of Peritonitis, or initial colon resection. Since there is existing evidence suggesting that conversion to open appendectomy is not only associated with complication status (perforation, abscess formation, and diffuse peritonitis, which were included in the definition of complicated appendicitis), but also with comorbidities and anatomical variants (retrocecal appendix), conversion was not included in the definition of complicated appendicitis to avoid bias^28^.

### Statistical analysis

Only the index hospitalization was used for all analyses, as no individual patient identifier is coded. To account for different comorbidity structures, we used the comorbidity score first introduced by Stausberg and colleagues^29^; validity has been affirmed in the German variant of the ICD-system.

The work has been reported in line with the strengthening the reporting of cohort, cross-sectional, and case–control studies in surgery (STROCSS) criteria^30^ (Supplemental Digital Content 2, http://links.lww.com/JS9/C473) and the strengthening the reporting of observational studies in epidemiology (STROBE) guidelines^31^. It was registered retrospectively with a Research Registry UIN (researchregistry9041) (https://researchregistry.knack.com/researchregistry#home/registrationdetails/64673ad2bd576a0027559076/). (not stated for peer review).

Odd’s ratios (OR) were calculated as risk assessment between the primary dependent variable, a CCE of ‘prolonged postoperative length of stay (>10 days, which was chosen based on the fact that no previous study has reported a longer average length of stay than nine days in case of complicated appendicitis), surgical site infection (ICD coding), interventional draining after surgery (OPS code), revision surgery (OPS code), ICU admission (‘treating unit coding’) (Supplementary Table 1, Supplemental Digital Content 1, http://links.lww.com/JS9/C472) and/or in-hospital mortality’ or in-hospital mortality as the secondary dependent variable and the primary independent variable TAS as well as secondary independent variables. Whether OR refers to CCE or in-hospital mortality will be stated where applicable in this analysis.

For the logistic regression model, the relationship between TAS and a CCE or in-hospital mortality was determined while accounting for possible confounders and the clustered data structure treating the constant hospital identifier as a random effect in an overall mixed effect model. Eligible confounding variables including comorbidity, age, and sex were included in the model. Likelihood tests were used to assess regression model accuracy. Sensitivity analyses included: changing the postoperative length of stay in the definition of the CCE, changing age intervals, changing time intervals of TAS, using different comorbidity scores, and analyzing relevant subgroups (age and sex). These adaptations did not lead to a differing overall result, while they did lead to absolute changes of resulting ORs. We excluded the presence of significant multicollinearities among confounding variables. The area under the receiver operating curve was obtained to test discrimination of the resulting logistic regression model. For result interpretation, an OR of <1·10 was considered clinically irrelevant. This was determined a priori.

Stata (Version 16; StataCorp LP) was used for all statistical analysis and data communication with the Federal Statistical Office. Values are stated as median with SD or median with interquartile range, where appropriate. *P*-values of ≤0·05 were considered significant.

### Identification of relevant research context

PubMed and MEDLINE were searched for existing evidence using the search terms ‘acute appendicitis’ and ‘timing of surgery’, which was last conducted on 1 November 2023, yielding a total of 356 results. All titles and abstracts were manually screened for relevance, resulting in 60 articles, which were then analyzed in detail. Only original clinical studies were included, except small (<500 cases) monocentric retrospective analyses, and all pediatric studies were excluded. Of the resulting 19 studies, a full text analysis was done, and all references were screened for relevance, yielding a final number of 22 original articles included as a reference. The remaining nine articles cited in the present article were individually chosen due to relevance in methods, introduction, and/or discussion.

## Results

### Study population

Between 1 January 2010 and 31 December 2021, 1 157 825 patient records from 1243 different hospitals were eligible for inclusion. In 286 764 cases, the recorded age was under 18 years and these records were dropped as defined by exclusion criteria. Of the remaining 871 061 records, 15 367 were defined as conservative and dropped for further analysis, leaving 855 694 adult patient records with surgical treatment in case of acute appendicitis in Germany in the stated time window (Fig. 1 and Supplementary Table 1, Supplemental Digital Content 1, http://links.lww.com/JS9/C472).

Baseline characteristics are shown in Table 1. 49·0% were female and the median age was 37 years. The median overall length of stay was 4 days. In 95·7% of cases, appendectomy was the only operative procedure and was performed via laparoscopy in 87·6% of cases. Complicated appendicitis by intraoperative finding mounted to 236 481 cases (27·6%). Postoperative length of stay was longer in complicated appendicitis (median 5·8 days vs. 2·9 days in uncomplicated appendicitis). The overall median time from admission to surgery (TAS) was 5·7 h. 52·2% of cases received surgery within 6 h after admission, while in 1·5% (13 021) of cases, surgery was performed longer than 72 h after admission (Table 1).

**Table 1:** Total cohort: patient characteristics.

	Overall	Uncomplicated appendicitis	Complicated appendicitis
Total no. of patients	855 694	619 213 (72·4 of all)	236 481 (27·6 of all)
Age (years; median, IQR)	37 (22·5–51·5)	32 (20–44)	53 (39·5–66·5)
≤49	588 297 (68·8)	485 080 (78·3)	103 217 (43·7)
50–69	193 760 (22·6)	105 338 (17·0)	88 422 (37·4)
>70	73 637 (8·6)	28 795 (4·7)	44 842 (19·0)
No. of females	418 821 (49·0)	314 924 (50·9)	103 897 (43·9)
Comorbidity score (mean, sd)^a^	100·2 (±2·4)	100·0 (±1·9)	100·6 (±3·3)
Postoperative length of stay (days; median, IQR)	3·3 (1·9–4·1)	2·9 (1·9–3·6)	5·8 (2·8–6·8)
Time from admission to surgery (hours; median, IQR)	5·7 (2·2–13·2)	5·7 (2·0–13·2)	5·7 (2·8–13·0)
Time from admission to surgery in detail^b^			
≤6 h	446 733 (52·2)	323 295 (52·2)	123 438 (52·3)
6–12 h	177 080 (20·7)	126 537 (20·4)	50 543 (21·4)
12–24 h	142 416 (16·7)	109 293 (17·7)	33 123 (14·0)
24–72 h	75 983 (8·9)	53 643 (8·7)	22 340 (9·5)
>72 h	13 021 (1·5)	6434 (1·0)	6587 (2·8)
Surgery during emergency hours: 6 pm through 6.59 am	303 551 (35·5)	212 570 (34·3)	90 981 (38·5)
Overall course			
Appendectomy only	818 977 (95·7)	618 321 (99·9)	220 656 (84·9)
Appendectomy with primary colon procedure or following revision surgery	36 717 (4·3)	892 (0·1)	35 825 (15·2)
Surgical details			
Laparoscopic approach	749 318 (87·6)	583 750 (94·3)	165 568 (70·0)
Open approach	54 782 (6·4)	30 215 (4·9)	24 567 (10·4)
Conversion	26 136 (3·1)	5075 (0·8)	21 061 (8·9)

Values in parentheses are percentages of the total in the patient group unless otherwise indicated.

a Comorbidity score first introduced by Stausberg and colleagues^27^, whose validity has been affirmed in the German variant of the ICD-system.

b Total of 855 233 due to 461 records without time coding of OPS procedures (3 dead, 0·7%). Surgical details refer to appendectomy (in 25 458, 3·0% of all cases, type of surgery was not coded and therefore unknown).

### Primary outcome

The CCE ‘prolonged postoperative length of stay (>10 days), surgical site infection, interventional draining after surgery, revision surgery, ICU admission, and/or mortality’ was observed in 8·0% of cases (68 400). It was more frequent in cases of complicated appendicitis (24·7 vs. 1·6% in uncomplicated appendicitis) and increased with age (Table 2, Supplementary Table 2, Supplemental Digital Content 1, http://links.lww.com/JS9/C472 and Supplementary Table 3, Supplemental Digital Content 1, http://links.lww.com/JS9/C472).

**Table 2:** Details for composite clinical endpoint by time from admission to surgery.

Time from admission to surgery ⇒	Up to 6 h after admission	6–12 h after admission	12–24 h after admission	24–72 h after admission	longer than 72 h after admission	*P*
Patients (855 233)	446 733 (100⇓, 52·2⇐)	177 080 (100⇓, 20·7⇐)	142 416 (100⇓, 16·7⇐)	75 983 (100⇓, 8·9⇐)	13 021 (100⇓, 1·5⇐)	
Uncomplicated app. (619 213, 72·4%⇑, 100%⇒)	323 295 (72·4⇑, 52·2⇐)	126 537 (71·5⇑, 20·4⇐)	109 293 (76·7⇑, 17·7⇐)	53 643 (70·6⇑, 8·7⇐)	6434 (49·4⇑, 1·0⇐)	<0·0001
Complicated app. (236 481, 27·6%⇑, 100%⇒)	123 438 (27·5⇑, 52·2⇐)	50 543 (28·5⇑, 21·4⇐)	33 123 (23·3⇑, 14·0⇐)	22 340 (29·4⇑, 9·5⇐)	6587 (50·6⇑, 2·8⇐)	<0·0001
Composite endpoint (68 400, 8·0%’)	32 401 (7·3)	14 677 (8·3)	10 065 (7·1)	7713 (10·2)	3544 (27·2)	<0·0001
Uncomplicated app. (10 002, 1·6%^a^)	5037 (1·6)	2110 (1·7)	1669 (1·5)	965 (1·8)	221 (3·4)	<0·0001
Complicated app. (58,398, 24·7%^a^)	27 364 (22·2)	12 567 (24·9)	8396 (25·4)	6748 (30·2)	3323 (50·5)	<0·0001
In-hospital mortality (1856, 0·2%’)	727 (0·2)	411 (0·2)	265 (0·2)	279 (0·4)	174 (1·3)	<0·0001
Uncomplicated app. (249, 0·04%^a^)	107 (0·03)	54 (0·04)	47 (0·04)	32 (0·06)	9 (0·14)	<0·0001
Complicated app. (1607, 0·68%^a^)	620 (0·5)	357 (0·7)	218 (0·7)	247 (1·1)	165 (2·5)	<0·0001
SSI (10 742, 1·3%’)	5487 (1·2)	2247 (1·3)	1428 (1·0)	1062 (1·4)	518 (4·0)	<0·0001
Uncomplicated app. (2564, 0·4%^a^)	1401 (0·4)	517 (0·4)	382 (0·4)	219 (0·4)	45 (0·7)	<0·0001
Complicated app. (8178, 3·5%^a^)	4086 (3·3)	1730 (3·4)	1046 (3·2)	843 (3·8)	473 (7·2)	<0·0001
Length of stay >10 days (41 541, 4·9%’)	18 763 (4·2)	8811 (5·0)	6544 (4·6)	5143 (6·8)	2280 (17·5)	<0·0001
Uncomplicated app. (5647, 0·9%^a^)	2507 (0·8)	1198 (1·0)	1080 (1·0)	690 (1·3)	172 (2·7)	<0·0001
Complicated app. (35 894, 15·2%^a^)	16 256 (13·2)	7613 (15·1)	5464 (16·5)	4453 (19·9)	2108 (32·0)	<0·0001
ICU admission (3898, 0·5%’)	2049 (0·5)	949 (0·5)	469 (0·3)	341 (0·5)	90 (0·7)	<0·0001
Uncomplicated app. (1910, 0·3%^a^)	1123 (0·4)	431 (0·3)	232 (0·2)	108 (0·2)	16 (0·3)	<0·0001
Complicated app. (1988, 0·8%^a^)	926 (0·8)	518 (1·0)	237 (0·7)	233 (1·0)	74 (1·1)	<0·0001
Drain after surgery (716, 0·1%’)	229 (0·1)	117 (0·1)	69 (0·1)	91 (0·1)	209 (1·6)	<0·0001
Uncomplicated app. (10, 0·0%^a^)	4 (0·0)	3 (0·0)	3 (0·0)	0	0	0·633
Complicated app. (705, 0·3%^a^)	225 (0·2)	114 (0·2)	66 (0·2)	91 (0·4)	209 (3·2)	<0·0001
Revision surgery (34 051, 4·0%’)	16 118 (3·6)	7309 (4·1)	4619 (3·2)	3820 (5·0)	2185 (16·8)	<0·0001
Uncomplicated app. (1793, 0·3%^a^)	983 (0·3)	371 (0·3)	269 (0·3)	140 (0·3)	30 (0·5)	0·001
Complicated app. (32 258, 13·7%^a^)	15 135 (12·3)	6938 (13·7)	4350 (13·1)	3680 (16·5)	2155 (32·7)	<0·0001

The composite clinical endpoint (third row) is equivalent to ‘at least one of those mentioned below’.

‘app’. for appendicitis. ICU admission was identified using admission codes to ICU departments in the patient records. ‘SSI’ for surgical site infection. Total of 855 233 due to 461 records without time coding of OPS procedures (3 dead, 0·7%). Length of stay refers to postoperative length of stay. Percentages in brackets in this row refer to the total cohort.

a Percentages in brackets in this row refer to the respective sub-cohort, that is, uncomplicated or complicated appendicitis. ⇒⇐ Percentages refer to row total, ⇑⇓ percentages refer to column total. *P*-values stem from chi-squared test (row). Composite endpoint short for composite clinical endpoint.

As TAS increased, the CCE was observed in a larger fraction of patients (within 6 h: 7·3%; 24–72 h: 10·2%; >72 h: 27·2%). In cases of uncomplicated appendicitis, an increase in the CCE (1·6 vs 3·4%) was observed in the group of patients receiving surgery after longer than 72 h. In complicated appendicitis, an increase in the CCE was observed after TAS of 6 h (22·2 vs. 24·9%, *P*<0·0001) (Table 2 and Supplementary Figure 1, Supplemental Digital Content 1, http://links.lww.com/JS9/C472).

In a univariable approach, TAS was associated with an increase of the CCE after 6 h (OR 1·16, 95% CI: 1·13–1·18) in the overall cohort (Table 3).

**Table 3:** Composite clinical endpoint and in-hospital mortality: univariable odd’s ratios and multivariable logistic regression model.

	Univariable odd’s ratio for composite endpoint			Multivariable odd’s ratio for composite endpoint		
Time from admission to surgery	Overall	Uncomplicated app.	Complicated app.	Overall	Uncomplicated app.	Complicated app.
≤6 h	1	1	1	1	1	1
6–12 h	1·16 [1·13–1·18, *P*<0·0001]	1·07 [1·02–1·13, *P*=0·008]	1·16 [1·13–1·19, *P*<0·0001]	1·04 [1·01–1·06, *P*=0·001]	1·00 [0·95–1·06, *P*=0·974]	1·07 [1·04–1·10, *P*<0·0001]
12–24 h	0·97 [0·95–1·0, *P*=0·018]	0·98 [0·93–1·04, *P*=0·474]	1·19 [1·16–1·23, *P*<0·0001]	1·04 [1·01–1·06, *P*=0·005]	1·07 [1·00–1·13, *P*=0·042]	1·18 [1·14–1·21, *P*<0·0001]
24–72 h	1·44 [1·41–1·48, *P*<0·0001]	1·16 [1·08–1·24, *P*<0·0001]	1·52 [1·47–1·57, *P*<0·0001]	1·32 [1·28–1·36, *P*<0·0001]	1·10 [1·02–1·19, *P*=0·013]	1·37 [1·33–1·42, *P*<0·0001]
>72 h	4·78 [4·59–4·98, *P*<0·0001]	2·25 [1·98–2·58, *P*<0·0001]	3·57 [3·40–3·76, *P*<0·0001]	3·42 [3·27–3·59, *P*<0·0001]	1·67 [1·43–1·94, *P*<0·0001]	2·96 [2·81–3·13, *P*<0·0001]
Surgery during regular hours: 7 am through 5.59 pm	1	1	1	1	1	1
Surgery during emergency hours: 6 pm through 6.59 am	1·28 [1·27–1·30, *P*<0·0001]	1·37 [1·32–1·43, *P*<0·0001]	1·12 [1·11–1·15, *P*<0·0001]	1·23 [1·20–1·25, *P*<0·0001]	1·37 [1·30–1·43, *P*<0·0001]	1·14 [1·11–1·16, *P*<0·0001]
	Univariable odd’s ratio for in-hospital mortality			Multivariable odd’s ratio for in-hospital mortality		
≤6 h	1	1	1	1	1	1
6–12 h	1·43 [1·26–1·61], *P*<0·0001	1·29 [0·93–1·79], *P*=0·128	1·41 [1·24–1·61], *P*<0·0001	1·06 [0·92–1·22, *P*=0·444]	0·83 [0·56–1·24, *P*=0·369]	1·09 [0·93–1·28, *P*=0·267]
12–24 h	1·14 [0·99–1·32], *P*=0·062	1·30 [0·92–1·83], *P*=0·134	1·31 [1·12–1·53], *P*=0·001	1·03 [0·87–1·22, *P*=0·711]	0·92 [0·60–1·41, *P*=0·706]	1·07 [0·89–1·29, *P*=0·486]
24–72 h	2·26 [1·97–2·60], *P*<0·0001	1·80 [1·21–2·68], *P*=0·003	2·21 [1·91–2·57], *P*<0·0001	1·04 [0·87–1·23, *P*=0·684]	0·58 [0·35–0·95, *P*=0·031]	1·12 [0·94–1·35, *P*=0·208]
>72 h	8·31 [7·04–9·81], *P*<0·0001	4·23 [2·14–8·36], *P*<0·0001	5·09 [4·28–6·05], *P*<0·0001	1·70 [1·37–2·11, *P*<0·0001]	0·89 [0·39–2·05, *P*=0·789]	1·72 [1·38–2·15, *P*<0·0001]
Surgery during regular hours: 7 am through 5.59 pm	1	1	1	1	1	1
Surgery during emergency hours: 6 pm through 6.59 am	1·56 [1·43–1·72, *P*<0·0001]	1·72 [1·35–2·22, *P*<0·0001]	1·37 [1·23–1·52, *P*<0·0001]	1·28 [1·14–1·45, *P*<0·0001]	1·49 [1·09–2·04, *P*=0·014]	1·22 [1·08–1·39, *P*=0·002]

‘app.’ for appendicitis. Composite clinical endpoint includes resurgery (relaparotomy, relaparoscopy), surgical site infection, postoperative length of stay over 10 days, admission to ICU, or death (absolute numbers in Table 2, and by age in Supplementary Table 2 (Supplemental Digital Content 1, http://links.lww.com/JS9/C472) and Supplementary Table 3 (Supplemental Digital Content 1, http://links.lww.com/JS9/C472). [ ] denote 95% CI for odd’s ratio estimators, ‘*P*’ short for *P*-value. ‘≤6 h’ and ‘Surgery during regular hours: 7 am through 5.59 pm’ were chosen as references, which is denoted stating ‘1’ in the respective rows. Multivariable odd’s ratios for age categories, sex, and comorbidity along with random effect (hospital identifier) logit estimators and area under the receiver operating curve in Supplementary Table 4 (Supplemental Digital Content 1, http://links.lww.com/JS9/C472). Composite endpoint short for composite clinical endpoint.

In the multivariable logistic regression approach, age, male sex, and comorbidity were significant factors associated with an increased CCE (Supplementary Table 4, Supplemental Digital Content 1, http://links.lww.com/JS9/C472). In this multivariable approach, TAS was significantly associated with a relevantly increased CCE after 24 h (OR, 1·32, 95% CI: 1·28–1·36) in the overall cohort. In a stratified analysis, the relevant increase in the CCE was observed after TAS of 12 h in cases of complicated appendicitis (OR 1·18, 95% CI: 1·14–1·21). After TAS of 24 h, this increase was noted in cases of uncomplicated appendicitis (OR 1·10, 95% CI: 1·02–1·19) (Table 3).

### Secondary outcomes

As a secondary endpoint, in-hospital mortality was analyzed. In-hospital death occurred in 1856 cases (0·2%), increased with age, and was higher in cases of complicated appendicitis (Table 2, Supplementary Table 2, Supplemental Digital Content 1, http://links.lww.com/JS9/C472, Supplementary Table 3, Supplemental Digital Content 1, http://links.lww.com/JS9/C472).

In uncomplicated appendicitis, a significant increase in in-hospital mortality was found after TAS of 72 h. In complicated appendicitis, an increase in in-hospital mortality was observed after TAS of 6 h (0·5 vs. 0·7%) (Table 2 and Supplementary Figure 1, Supplemental Digital Content 1, http://links.lww.com/JS9/C472).

In a stratified analysis, in-hospital mortality increased significantly after TAS of 72 h in cases of complicated appendicitis (OR 1·62, 95% CI: 1·31–2·03), which was not observed in cases of uncomplicated appendicitis (OR 0·89, 95% CI: 0·39–2·05) (Table 3).

**Figure 2:**
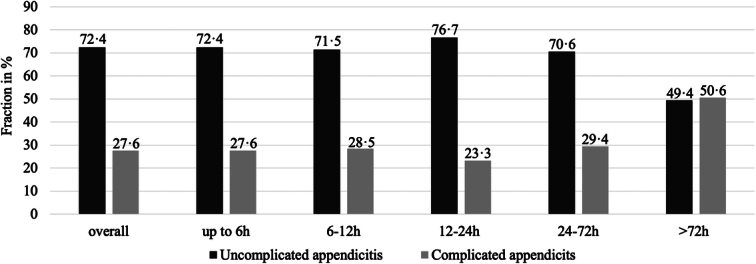
Fraction of complicated and uncomplicated appendicitis by time from admission to surgery. Details in Table 1. Numbers represent percentages.

As another secondary endpoint, the fraction of uncomplicated to complicated appendicitis by intraoperative finding was stable (overall 72·4 vs. 27·6%, 2·6:1) up to TAS of 72 h (70·6 vs. 29·4%, 2·4:1). After 72 h, the fraction of complicated appendicitis increased (49·4 vs. 50·6%, 0·98:1) (Table 2 and Fig. 2). The ratio of uncomplicated to complicated appendicitis was associated with age (Supplementary Table 2, Supplemental Digital Content 1, http://links.lww.com/JS9/C472, Supplementary Table 3, Supplemental Digital Content 1, http://links.lww.com/JS9/C472 and Supplementary Figure 2, Supplemental Digital Content 1, http://links.lww.com/JS9/C472).

### Surgery during emergency hours and factors associated with delayed surgery

The primary and secondary endpoints were stratified by regular time surgery (7 am–5.59 pm) and emergency hours (6 pm–6.59 am) in Supplementary Table 5 (Supplemental Digital Content 1, http://links.lww.com/JS9/C472). In case of surgery during emergency hours, a higher frequency of complicated cases (30 vs. 26·3%) was noted with a higher fraction in case of TAS of 6 h (59·7 vs. 48·2%) and more adverse outcome events in both complicated and uncomplicated appendicitis (overall CCE 9·2 vs. 7·2%) (Supplementary Table 5, Supplemental Digital Content 1, http://links.lww.com/JS9/C472). In the multivariable logistic regression, this association was strongest for cases of uncomplicated appendicitis (OR for the CCE 1·37, 95% CI: 1·30–1·43) (Table 3).

Risk factors associated with delayed surgery, that is, TAS longer than 12 h in uncomplicated and TAS longer than 24 h in complicated appendicitis were analyzed by patient factors. In a multivariable approach, age, female sex, night-time admission, weekend admission, a known previous surgery, obesity, and therapeutic anticoagulation were significantly associated with delayed performance of surgery (Supplementary Table 6, Supplemental Digital Content 1, http://links.lww.com/JS9/C472).

## Discussion

This comprehensive, nationwide study of acute appendicitis in a 12-year time window demonstrated no clinically relevant increase of a CCE if surgery was performed within 24 h after admission in cases of uncomplicated appendicitis with a threshold of OR ≥1·10. In complicated appendicitis, TAS longer than 12 h was associated with an increase of a CCE. After 72 h, as secondary endpoints, higher in-hospital mortality was found, and no overall increased rate of complicated appendicitis after TAS of up to 72 h was observed.

So far, guidelines do not distinguish between primarily presumed uncomplicated and complicated appendicitis regarding a recommended time window of surgery; the current state of the art in managing acute appendicitis is appendectomy. The recommendation for a specific time frame for surgery assumes that immediate surgical treatment reduces complications, especially perforation, which contrasts with the idea that uncomplicated cases may not progress to complicated appendicitis.

A prospective randomized controlled trial studied delaying surgery after 6 am in comparison with immediate surgery. The primary outcome (30-day-postsurgery complication rate) showed noninferiority of delaying appendectomy (mean delay 6·6 h) for 127 patients presenting between 2 am and 4 am^32^, which is in alignment with our results.

Existing evidence states no association with night-time (and therefore emergency) surgery and complication rates in appendectomies^22–25^. In this analysis, a slightly increased fraction of complicated appendicitis was found during emergency hours (30.0% of emergency hour appendectomies, while the overall fraction was 27.6%), which might be due to severity of symptoms at the time of presentation. However, independent of complication status, that is, after stratification, surgery during emergency hours was associated with higher observed fractions of the CCE and in-hospital mortality. During emergency circumstances, patient and organizational factors must be taken into account to explain these higher adverse outcome rates.

Based on the CCE and weighing ORs for an increased likelihood of the CCE, results of the present analysis stratified by age and signs of complicated appendicitis are represented in Supplementary Figure 3 (Supplemental Digital Content 1, http://links.lww.com/JS9/C472). As a result of weighing ORs for increased likelihood of the CCE, appendectomy during emergency hours should carefully be weighed and only be performed in case of clinical urgency. Since performance of surgery during emergency hours in complicated appendicitis was associated with an increased OR of the CCE, the results of this study indicate a benefit of surgery performance during regular hours if this can be achieved with a delay of no more than 6 h. Also, risk factors associated with a delay of surgery, which were identified in this study, should be assessed at time of presentation.

The strength of this analysis is the completeness of data, which allows for a real-world analysis of more than 850 000 cases with available information of TAS. In comparison to the APPAC trial^3^, in which 337 patients out of 1379 were found to have complicated appendicitis (24·4%), the frequency of complicated appendicitis is in close range with our definition identifying 27·6% to be complicated cases. Complication rates of 20·5% in the APPAC trial differ from an observed rate of 3% of serious adverse rates in the CODA^4^ trial, which is a matter of definition and lies within range of the 8·0% of CCE, which was observed in this analysis. With the overall aim of optimizing quality of surgical care, it is pivotal to identify risk factors associated with morbidity and mortality, like time from admission to surgery in case of complicated appendicitis in this analysis. This data may be particularly relevant for settings equipped with restricted medical resources, in which case clinicians must weigh the benefits and risks of different therapeutic approaches, considering individual characteristics and patient preferences. We consider the results of this analysis in line with pre-existing evidence describing an association with delay of surgery and adverse outcome in appendicitis^7–17^, while differences in study size, methodology, and endpoint definition have to be considered, and it adds to existing literature a stratified view on uncomplicated vs complicated appendicitis.

This study has some limitations. First, due to its retrospective nature, no conclusions on causality can be drawn. Possible explanations for in-hospital delay like a primarily suspected differential diagnosis with a conservative treatment attempt could not be identified if acute appendicitis was coded as main diagnosis. As another limitation, no clinical data on implemented scores, blood count or fever were available. Therefore, no adjustment for medical urgency according to these parameters was possible, which might introduce bias in cases receiving surgery during emergency hours. Additionally, there was no specific coding of the time from symptom onset until hospital admission in the patient history, even though this parameter is highly subjective. Also, data on readmissions were unavailable. No stratification for pregnancy was conducted. Identification of complication status was based on ICD and procedure coding of the initial procedure and is impossible at the time of presentation of an individual patient. However, because mortality and a CCE were increased in the case of complicated appendicitis, this status seemed to be a relevant parameter impacting the outcome beyond the initial surgery and independently of comorbidity and age. Risk factors for complicated appendicitis, which can be assessed at the time of presentation, have previously been identified. In addition to these limitations, surgical site infection is a subjective side diagnosis coded by coding staff or physicians with no clear underlying definition, introducing bias in this aspect of the CCE. Coding of the stratification between complicated and uncomplicated appendicitis was based on procedural codes, which are specific to the German health system. It was also partly based on ICD-10 coding in a German variation; therefore, and for other reasons unreflected by available variables, generalizability may be restricted to health care systems comparable to that in Germany. The finding of increased CCE and in-hospital mortality during emergency hour appendectomy, which is in contrast to some of the existing evidence, might only be partly translatable to other health-care systems, possibly due to structural circumstances in German hospitals and/or patient comorbidity structures. In this analysis, no information on surgeon experience or expertise was available, which may be a confounder with regards to emergency surgery as a structural effect in Germany^22^.

Our analysis, in addition to growing evidence, challenges the routine perception of acute uncomplicated appendicitis to proceed to a complicated form up to 72 h. With respect to the increase in a CCE and mortality in cases of complicated appendicitis after 12 and 72 h, respectively, relative urgency in this sub-cohort is acknowledged. Our results revealed a clear distinction between the two entities, complicated and uncomplicated appendicitis, in terms of risk for the CCE and in-hospital mortality, indicating differing time windows for surgical therapy. It therefore seems crucial to identify patients with high risk of complicated appendicitis to triage surgical urgency. Independent of complication status, performance of surgery during emergency hours was associated with a higher risk of CCE and in-hospital mortality.

In conclusion, this work found an increase of a clinical endpoint after in-hospital delay of 12 h for complicated appendicitis and after 24 hours for uncomplicated appendicitis. In these time windows, the proportion of complicated appendicitis among all cases was stable. Both the clinical endpoint and in-hospital mortality were increased if appendectomy was performed during emergency hours. Age, female sex, night-time admission, weekend admission, a known previous surgery, obesity, and therapeutic anticoagulation were identified as patient and circumstantial factors associated with delayed performance of surgery.

## Ethical approval

No ethical vote was necessary for this large-scale, nationwide cohort analysis, as defined by German data safety law.

## Consent

No individual patient consent was necessary for this large-scale, nationwide cohort analysis. No individual patient data was accessed or analyzed in detail.

## Sources of funding

This publication is funded by the ‘Open Access’ program of the Deutsche Forschungsgemeinschaft, DFG. The funder had no role in study design, data collection and analysis, decision to publish, or preparation of the manuscript.

## Author contribution

K.U.: conceptualized the project, conducted all data curation and relevant formal analyses in close contact with the Federal Statistical Office Germany, and was in charge of methodology and editing the manuscript (Writing – original draft, and Writing – review and editing). J.D. and P.B.: assisted in methodology, data curation and formal data analysis, and in writing – review and editing; D.S. and C.T.G.: revised (Writing – review and editing) the manuscript and were very significant in supervision and in the discussion of clinically relevant aspects of the manuscript; A.W.: helped conceptualize the project, supervised the project, and overviewed the process of all formal analyses and editing (Writing – review and editing).

## Conflicts of interest disclosure

The authors declare that they have no financial conflict of interest with regard to the content of this report.

## Research registration unique identifying number (UIN)

(researchregistry9041) (https://researchregistry.knack.com/researchregistry#home/registrationdetails/64673ad2bd576a0027559076/).

## Guarantor

Konstantin Uttinger and Armin Wiegering are guarantors for this analysis.

## Data sharing statement

Since all data accessing and analysis steps were conducted in collaboration with the Federal Statistical Office in Germany, Gustav-Stresemann-Ring 11, 65189 Wiesbaden, represented by their president, Dr. Ruth Brand, and since all data are protected by their guidelines regarding highly sensitive patient-record data, no individual data can be made available. However, in accordance with their guidelines, all data can be permanently accessed after acceptance of an application for data usage by the Federal Statistical Office. Using the inclusion criteria, the same patient cohort as used for this analysis can be identified. It is possible to contact the corresponding author of this Study, Dr. Konstantin Uttinger, to request access to the Stata protocol for a detailed analysis of the coding steps for identification of the study cohort.

## Provenance and peer review

Not commissioned, externally peer-reviewed.

## Assistance with the study

None.

## Presentation

None.

## Supplementary Material
