# Supplementary material for: The impact of surgical timing on outcome in acute appendicitis in adults: a retrospective observational population-based cohort study
Source: Int J Surg. 2024 May 3;110(8):4850–8. doi: 10.1097/JS9.0000000000001528 (PMC11325913; doi:10.1097/JS9.0000000000001528)
Supplement: Supplementary file 1 [file js9-110-4850-s001.docx]

# Supplements

| **ICD Diagnoses** |
| --- |
| Main diagnosis:  Appendicitis (K35), subgroup “unclear” (none of the following) or K35.8 (not otherwise specified), without perforation (K35.30), with perforation (K35.31), with abscess formation (K35.32), with generalized peritonitis (K35.2)  Side diagnoses:  Peritonitis (K65)  Surgical site infection: T81.4 |
| **OPS Codes** |
| Appendectomy: 5-470 with all subgroups, 5-479  Colon procedures: 5-455  Interventional drain: 8-146  Relaparoscopy: 1-694, 5-479.0 (the latter only of this was not the primary procedure)  Relaparotomy: 5-541.2, 5-541.3, 5.467.5x |

**Supp. Table 1: ICD diagnoses and OPS codes for patient identification.**

|  | **up to 6 hours after admission** | **6-12 hours after admission** | **12-24 hours after admission** | **24-72 hours after admission** | **longer than 72 hours after admission** | p-value |
| --- | --- | --- | --- | --- | --- | --- |
| **Patients** (855,233) | 446,733 (52·2) | 177,080 (20·7) | 142,416 (16·7) | 75,983 (8·9) | 13,021 (1·5) |  |
| **Composite endpoint** (68,400, 8·0%) | 32,401 (7·3) | 14,677 (8·3) | 10,065 (7·1) | 7,713 (10·2) | 3,544 (27·2) | <0·0001 |
| Mortality (1,856, 0·2%) | 727 (0·2) | 411 (0·2) | 265 (0·2) | 279 (0·4) | 174 (1·3) | <0·0001 |
| **Age ≤ 49** (588,297, 68·8%) | 305,256 (68·3) | 118,423 (66·9) | 104,589 (73·4) | 52,845 (69·6) | 6,878 (52·8) |  |
| Composite endpoint (23,823, 4·1%) | 12,133 (4·0) | 4,764 (4·0) | 3,636 (3·5) | 2,390 (4·5) | 900 (13·1) | <0·0001 |
| Mortality (49, 0·0%) | 25 (0·0) | 11 (0·0) | 7 (0·0) | 3 (0·0) | 3 (0·0) | 0·024 |
| **Age 50-59** (118,432, 13·8%) | 65,394 (14·6) | 25,150 (14·2) | 16,806 (11·8) | 9,106 (12·0) | 1,908 (14·7) |  |
| Composite endpoint (12,735, 10·8%) | 6,327 (9·7) | 2,778 (11·1) | 1,743 (10·4) | 1,280 (14·1) | 607 (31·8) | <0·0001 |
| Mortality (103, 0·1%) | 54 (0·1) | 29 (0·1) | 8 (0·1) | 9 (0·1) | 2 (0·1) | 0·220 |
| **Age 60-69** (75,328, 8·8%) | 40,902 (9·2) | 16,716 (9·4) | 10,037 (7·1) | 6,024 (7·9) | 1,612 (12·4) |  |
| Composite endpoint (11,756, 15·6%) | 5,673 (13·9) | 2,610 (15·6) | 1,582 (15·8) | 1,239 (20·6) | 652 (40·5) | <0·0001 |
| Mortality (221, 0·3%) | 102 (0·3) | 49 (0·3) | 24 (0·2) | 27 (0·5) | 19 (1·2) | <0·0001 |
| **Age 70-79** (50,548, 5·9%) | 24,966 (5·6) | 11,515 (6·5) | 7,327 (5·1) | 5,042 (6·6) | 1,663 (12·8) |  |
| Composite endpoint (11,906, 23·6%) | 5,058 (20·3) | 2,669 (23·2) | 1,789 (24·4) | 1,561 (31·0) | 829 (49·9) | <0·0001 |
| Mortality (555, 1·1%) | 215 (0·9) | 120 (1·0) | 76 (1·0) | 70 (1·3) | 73 (4·4) | <0·0001 |
| **Age 80-89** (20,693, 2·4%) | 9,231 (2·1) | 4,712 (2·7) | 3,239 (2·3) | 2,622 (3·5) | 875 (6·7) |  |
| Composite endpoint (7,100, 34·3%) | 2,788 (30·2) | 1,609 (34·2) | 1,130 (34·9) | 1,075 (41·0) | 498 (56·9) | <0·0001 |
| Mortality (695, 3·4%) | 249 (2·7) | 144 (3·1) | 110 (3·4) | 123 (4·7) | 68 (7·8) | <0·0001 |
| **Age >90** (2,396, 0·3%) | 984 (0·2) | 564 (0·3) | 418 (0·3) | 344 (0·5) | 85 (0·7) |  |
| Composite endpoint (1,080, 45·1%) | 422 (42·9) | 247 (43·8) | 185 (44·3) | 168 (48·8) | 58 (68·2) | <0·0001 |
| Mortality (236, 9·9%) | 82 (8·3) | 58 (10·3) | 40 (9·6) | 47 (13·7) | 9 (10·6) | 0·078 |

**Supp. Table 2: Mortality and composite clinical endpoint in subgroups by time from admission to surgery.** Composite clinical endpoint includes resurgery (relaparotomy, relaparoscopy), surgical site infection, postoperative length of stay over ten days, admission to intensive care unit, or death. Total of 855,233 due to 461 records without time coding of OPS procedures (3 dead, 0·7%). Composite endpoint short for composite clinical endpoint.

|  | **Uncomplicated appendicitis** | **Complicated appendicitis** |
| --- | --- | --- |
| **Overall number of patients** (855,694) | 619,213 (72·4 of all) | 236,481 (27·6 of all) |
| **Composite endpoint** (68,861, 8·1%) | 10,013 (1·6) | 58,848 (24·9) |
| **Mortality** (1,859, 0·2%) | 249 (0·04) | 1,610 (0·68) |
| **Age in groups in detail** |  |  |
| **Age ≤ 49** (588,297, 68·8%) | 485,080 (78·3) | 103,217 (43·7) |
| Composite endpoint (24,129, 4·1%) | 5,099 (1·1) | 19,030 (18·4) |
| Mortality (49, 0·0%) | 8 (0·0) | 41 (0·04) |
| **Age 50-59** (118,432, 13·8%) | 68,795 (11·1) | 49,637 (21·0) |
| Composite endpoint (12,803, 10·8%) | 1,362 (2·0) | 11,441 (23·1) |
| Mortality (103, 0·1%) | 15 (0·02) | 88 (0·18) |
| **Age 60-69** (75,328, 8·8%) | 36,543 (5·9) | 38,785 (16·4) |
| Composite endpoint (11,793, 15·7%) | 1,134 (3·1) | 10,659 (27·5) |
| Mortality (221, 0·3%) | 21 (0·06) | 200 (0·52) |
| **Age 70-79** (50,548, 5·9%) | 20,811 (3·4) | 29,737 (12·6) |
| Composite endpoint (11,941, 23·6%) | 1,305 (6·3) | 10,636 (35·8) |
| Mortality (555, 1·1%) | 70 (0·34) | 485 (1·63) |
| **Age 80-89** (20,693, 2·4%) | 7,241 (1·2) | 13,452 (5·7) |
| Composite endpoint (7,114, 34·4%) | 956 (13·2) | 6,158 (45·8) |
| Mortality (695, 3·4%) | 98 (1·4) | 597 (4·44) |
| **Age >90** (2,396, 0·3%) | 743 (0·1) | 1,653 (0·7) |
| Composite endpoint (1,081, 45·1%) | 157 (21·1) | 924 (55·9) |
| Mortality (236, 9·9%) | 37 (5·0) | 199 (12·04) |

**Supp. Table 3: In-hospital outcome for complicated vs. complicated appendicitis by age.** Composite clinical endpoint includes resurgery (relaparotomy, relaparoscopy), surgical site infection, postoperative length of stay over ten days, admission to intensive care unit, or death. Composite endpoint short for composite clinical endpoint.

**Supp. Figure 1: Composite clinical endpoint and in-hospital mortality by time from admission to surgery.** Data are based on descriptive fractions. Details in Table 2. P-values compare fraction of a composite clinical endpoint (CCE) or mortality by chi-squared test to previous group of in-hospital delay. “mort” for in-hospital mortality.

|  | **Univariable** Odd’s Ratio for **composite endpoint** | | | **Multivariable** Odd’s Ratio for **composite endpoint** | | |
| --- | --- | --- | --- | --- | --- | --- |
|  | Overall | Uncomplicated app. | Complicated app. | Overall _1_ | Uncomplicated app. _2_ | Complicated app. _3_ |
| **Age (years)** |  |  |  |  |  |  |
| ≤ 49 | 1 | 1 | 1 | 1 | 1 | 1 |
| 50–59 | 2·83 [2·77-2·90, p<0·0001] | 1·90 [1·79-2·02, p<0·0001] | 1·33 [1·29-1·36, p<0·0001] | 3·11 [3·03-3·18, p<0·0001] | 2·16 [2·03-2·30, p<0·0001] | 1·46 [1·42-1·50, p<0·0001] |
| 60-69 | 4·34 [4·24-4·44, p<0·0001] | 3·01 [2·82-3·22, p<0·0001] | 1·68 [1·63-1·72, p<0·0001] | 4·61 [4·49-4·72, p<0·0001] | 3·38 [3·16-3·62, p<0·0001] | 1·80 [1·75-1·86, p<0·0001] |
| 70-79 | 7·23 [7·06-7·41, p<0·0001] | 6·30 [5·92-6·70, p<0·0001] | 2·46 [2·39-2·53, p<0·0001] | 6·56 [6·39-6·73, p<0·0001] | 6·50 [6·07-6·95, p<0·0001] | 2·31 [2·24-2·39, p<0·0001] |
| 80-89 | 12·25 [11·87-12·64, p<0·0001] | 14·32 [13·30-15·41, p<0·0001] | 3·73 [3·60-3·88, p<0·0001] | 8·84 [8·53-9·16, p<0·0001] | 12·26 [11·28-13·32, p<0·0001] | 2·90 [2·78-3·02, p<0·0001] |
| >89 | 19·22 [17·72-20·85, p<0·0001] | 25·22 [21·10-30·14, p<0·0001] | 5·61 [5·08-6·19, p<0·0001] | 11·58 [10·56-12·71, p<0·0001] | 17·79 [14·48-21·85, p<0·0001] | 3·66 [3·29-4·08, p<0·0001] |
| **Sex** |  |  |  |  |  |  |
| female | 1 | 1 | 1 | 1 | 1 | 1 |
| male | 1·23 [1·21-1·25, p<0·0001] | 1·34 [1·28-1·39, p<0·0001] | 0·98 [0·96-1·00, p=0·075] | 1·25 [1·23-1·28, p<0·0001] | 1·38 [1·32-1·44, p<0·0001] | 1·02 [1·00-1·04, p=0·037] |
| **Comorbidity score *** (incremental) | 1·29 [1·28-1·29, p<0·0001] | 1·26 [1·25-1·27, p<0·0001] | 1·31 [1·28-1·32, p<0·0001] | 1·22 [1·22-1·23, p<0·0001] | 1·22 [1·21-1·23, p<0·0001] | 1·17 [1·17-1·18, p<0·0001] |
|  | **Univariable** Odd’s Ratio for **in-hospital mortality** | | | **Multivariable** Odd’s Ratio for **in-hospital mortality** | | |
|  | Overall | Uncomplicated app. | Complicated app. | Overall _4_ | Uncomplicated app. _5_ | Complicated app. _6_ |
| **Age (years)** |  |  |  |  |  |  |
| ≤ 49 | 1 | 1 | 1 | 1 | 1 | 1 |
| 50–59 | 10·45 [7·44-14·68], p<0·0001 | 13·22 [5·61-31·19], p<0·0001 | 4·47 [3·08-6·48], p<0·0001 | 7·46 [5·25-10·60, p<0·0001] | 12·29 [5·17-29·26, p<0·0001] | 3·60 [2·44-5·30, p<0·0001] |
| 60-69 | 35·32 [25·92-48·14], p<0·0001 | 34·86 [15·44-78·72], p<0·0001 | 13·04 [9·32-18·26], p<0·0001 | 17·46 [12·65-24·10, p<0·0001] | 22·92 [9·86-53·31, p<0·0001] | 7·79 [5·48-11·07, p<0·0001] |
| 70-79 | 133·27 [99·50-178·51], p<0·0001 | 204·64 [98·46-425·32], p<0·0001 | 41·72 [30·33-57·40], p<0·0001 | 38·42 [28·33-52·10, p<0·0001] | 74·49 [34·79-159·49, p<0·0001] | 15·56 [11·15-21·71, p<0·0001] |
| 80-89 | 417·22 [312·18-557·60], p<0·0001 | 831·88 [404·49-1,710·86], p<0·0001 | 116·87 [85·12-160·45], p<0·0001 | 79·83 [58·96-108·09, p<0·0001] | 170·85 [80·27-363·65, p<0·0001] | 31·26 [22·42-43·57, p<0·0001] |
| >89 | 1,311·66 [961·48-1,789·39], p<0·0001 | 3,177·70 [1,474·62-6,847·74], p<0·0001 | 344·42 [245·12-483·94], p<0·0001 | 247·85 [177·60-345·87, p<0·0001] | 450·59 [193·68-1,049·28, p<0·0001] | 98·94 [68·63-142·64, p<0·0001] |
| **Sex** |  |  |  |  |  |  |
| female | 1 | 1 | 1 | 1 | 1 | 1 |
| male | 1·48 [1·35-1·63], p<0·0001 | 1·42 [1·10-1·83], p=0·006 | 1·24 [1·12-1·37], p<0·0001 | 1·28 [1·14-1·43, p<0·0001] | 1·34 [0·99-1·82, p=0·059] | 1·25 [1·10-1·41, p<0·0001] |
| **Comorbidity score *** (incremental) | 1·49 [1·47-1·50], p<0·0001 | 1·68 [1·64-1·71], p<0·0001 | 1·39 [1·38-1·40], p<0·0001 | 1·37 [1·35-1·38, p<0·0001] | 1·47 [1·43-1·52, p<0·0001] | 1·33 [1·32-1·35, p<0·0001] |

**Supp. Table 4: Composite clinical endpoint and in-hospital mortality: univariable Odd’s Ratios and multivariable logistic regression model.** “app.” for appendicitis. Results for time from admission to surgery as a categorical variable and surgery by time of performance (emergency vs non-emergency) from the same model in Table 3. Composite clinical endpoint includes resurgery (relaparotomy, relaparoscopy), surgical site infection, postoperative length of stay over ten days, admission to intensive care unit, or death. *Comorbidity score first introduced by Stausberg and colleagues^27^, whose validity has been affirmed in the German variant of the ICD-system. “≤ 49“ and “female“ were chosen as references, which is denoted stating “1” in the respective rows. [ ] denote 95% Confidence Intervals for Odd’s Ratio estimators, “p” short for p-value. _n_ for model discrimination for the following, in which the logit estimate refers to the estimator of the random effect (treating hospital): Model 1: Logit estimate 0·47, 95% Confidence-Interval (95 % CI) 0·45-0·49, area under the Receiving Operator Curve: 0·71, 95 % CI 0·71-0·72, model 2: Logit estimate 0·62, 95% CI 0·59-0·66, area under the Receiving Operator Curve: 0·79, 95 % CI 0·79-0·80, model 3: Logit estimate 0·47, 95% CI 0·45-0·49, area under the Receiving Operator Curve: 0·71, 95 % CI 0·71-0·72, model 4: Logit estimate 0·64, 95% CI 0·56-0·74, area under the Receiving Operator Curve: 0·99, 95 % CI 0·99-0·99, model 5: Logit estimate 0·83, 95% CI 0·55-1·26, area under the Receiving Operator Curve: 0·99, 95 % CI 0·99-1·0, model 6: Logit estimate 0·64, 95% CI 0·55-0·74, area under the Receiving Operator Curve: 0·98, 95 % CI 0·97-0·98· Composite endpoint short for composite clinical endpoint.

**Supp. Figure 2: Fraction of complicated and uncomplicated appendicitis by age.** Details in Supp. Table 3.

|  | **Uncomplicated appendicitis** | **Complicated appendicitis** |
| --- | --- | --- |
| **Surgery during regular hours: 7 am through 5.59 pm** (551,682, 64·5%) | 406,632 (73·7) | 145,050 (26·3) |
| **Composite endpoint** (40,461, 7·3%) |  |  |
| **Mortality** (998, 0·2%) | 131 (0·03) | 867 (0·6) |
| **Time to first procedure in detail** |  |  |
| ≤ **6 h** (265,655, 48·2%) | 194,018 (47·7) | 71,637 (49·4) |
| Composite endpoint (17,586, 6·6%) | 2,577 (1·3) | 15,009 (21·0) |
| Mortality (333, 0·13%) | 53 (0·03) | 280 (0·39) |
| **6-12 h** (92,805, 16·8%) | 68,656 (16·9) | 24,149 (16·7) |
| Composite endpoint (6,163, 6·6%) | 999 (1·5) | 5,164 (21·4) |
| Mortality (143, 0·15%) | 14 (0·02) | 129 (0·53) |
| **12-24** **h** (122,037, 22·1%) | 94,752 (23·3) | 27,285 (18·8) |
| Composite endpoint (8,157, 6·7%) | 1,371 (1·5) | 6,786 (24·9) |
| Mortality (209, 0·17%) | 38 (0·04) | 171 (0·63) |
| **24-72 h** (60,283, 10·9%) | 43,635 (10·7) | 16,648 (11·5) |
| Composite endpoint (5,722, 9·5%) | 725 (1·7) | 4,997 (30·0) |
| Mortality (184, 0·31%) | 19 (0·04) | 165 (0·99) |
| **> 72 h** (10,902, 2·0%) | 5,571 (1·4) | 5,331 (3·7) |
| Composite endpoint (2,833, 26·0%) | 166 (3·0) | 2,667 (50·0) |
| Mortality (129, 1·2%) | 7 (0·13) | 122 (2·3) |
| **Surgery during emergency hours: 6 pm through 6.59 am** (303,551, 35·5%) | 212,570 (70·0) | 90,981 (30·0) |
| **Composite endpoint** (27,939, 9·2%) |  |  |
| **Mortality** (858, 0·3%) | 118 (0·06) | 740 (0·8) |
| **Time to first procedure in detail** |  |  |
| ≤ **6 h** (181,078, 59·7%) | 129,277 (60·8) | 51,801 (56·9) |
| Composite endpoint (14,815, 8·2%) | 2,460 (1·9) | 12,355 (23·9) |
| Mortality (394, 0·22%) | 54 (0·04) | 340 (0·66) |
| **6-12 h** (84,275, 27·8%) | 57,881 (27·2) | 26,394 (29·0) |
| Composite endpoint (8,514, 10·1%) | 1,111 (1·9) | 7,403 (28·1) |
| Mortality (268, 0·32%) | 40 (0·07) | 228 (0·86) |
| **12-24** **h** (20,379, 6·7%) | 14,541 (6·8) | 5,838 (6·4) |
| Composite endpoint (1,908, 9·4%) | 298 (2·1) | 1,610 (27·6) |
| Mortality (56, 0·27%) | 9 (0·06) | 47 (0·81) |
| **24-72 h** (15,700, 5·2%) | 10,008 (4·7) | 5,692 (6·3) |
| Composite endpoint (1,991, 12·7%) | 240 (2·4) | 1,751 (30·8) |
| Mortality (95, 0·61%) | 13 (0·13) | 82 (1·4) |
| **> 72 h** (2,119, 0·7%) | 863 (0·4) | 1,256 (1·4) |
| Composite endpoint (711, 33·6%) | 55 (6·4) | 656 (52·2) |
| Mortality (45, 2·1%) | 3 (0·3) | 42 (3·3) |

**Supp. Table 5: In-hospital mortality for complicated vs. complicated appendicitis by time of day of surgery.** Composite clinical endpoint includes resurgery (relaparotomy, relaparoscopy), surgical site infection, postoperative length of stay over ten days, admission to intensive care unit, or death. Total of 855,233 due to 461 records without time coding of OPS procedures (3 dead, 0·7%). Composite endpoint short for composite clinical endpoint.

|  | **Univariable** Odd’s Ratio for **delayed surgery** | 95% Confidence-Interval, p-value | **Multivariable** Odd’s Ratio for **delayed surgery** | 95% Confidence-Interval, p-value |
| --- | --- | --- | --- | --- |
| **Age (years)** |  |  |  |  |
| ≤ 49 | 1 | 1 | 1 | 1 |
| 50–59 | 1·61 | 1·59-1·64, p<0·0001 | 1·67 | 1·64-1·69, p<0·0001 |
| 60-69 | 2·02 | 1·98-2·05, p<0·0001 | 2·07 | 2·03-2·11, p<0·0001 |
| 70-79 | 2·76 | 2·71-2·81, p<0·0001 | 2·66 | 2·61-2·72, p<0·0001 |
| 80-89 | 3·67 | 3·57-3·78, p<0·0001 | 3·08 | 2·98-3·18, p<0·0001 |
| >89 | 4·30 | 3·97-4·67, p<0·0001 | 3·19 | 2·93-3·47, p<0·0001 |
| **Sex** |  |  |  |  |
| female | 1 | 1 | 1 | 1 |
| male | 0·88 | 0·87-0·89, p<0·0001 | 0·85 | 0·84-0·86, p<0·0001 |
| **Comorbidity score *** (incremental) | 1·10 | 1·10-1·11, p<0·0001 | 1·09 | 1·09-1·10, p<0·0001 |
| **Time of admission** |  |  |  |  |
| 0.00 am through 5 am | 1 | 1 | 1 | 1 |
| 5.01 am through 4 pm | 0·68 | 0·67-0·69, p<0·0001 | 0·64 | 0·63-0·65, p<0·0001 |
| 4.01 pm through 23.59 pm | 0·65 | 0·64-0·66, p<0·0001 | 0·62 | 0·61-0·64, p<0·0001 |
| **Day of admission** |  |  |  |  |
| Sunday | 1 | 1 | 1 | 1 |
| Monday | 0·95 | 0·94-0·97, p<0·0001 | 0·97 | 0·95-0·99, p=0·003 |
| Tuesday | 0·91 | 0·89-0·93, p<0·0001 | 0·93 | 0·91-0·95, p<0·0001 |
| Wednesday | 0·88 | 0·86-0·90, p<0·0001 | 0·89 | 0·87-0·91, p<0·0001 |
| Thursday | 0·80 | 0·79-0·82, p<0·0001 | 0·81 | 0·79-0·82, p<0·0001 |
| Friday | 0·78 | 0·77-0·80, p<0·0001 | 0·78 | 0·76-0·79, p<0·0001 |
| Saturday | 0·99 | 0·97-1·01, p=0·259 | 0·98 | 0·96-1·00, p=0·073 |
| **Known previous surgery** |  |  |  |  |
| No known previous surgery | 1 | 1 | 1 | 1 |
| Known previous surgery | 2·31 | 2·21-2·41, p<0·0001 | 1·44 | 1·37-1·51, p<0·0001 |
| **Obesity** |  |  |  |  |
| No obesity | 1 | 1 | 1 | 1 |
| Obesity | 1·38 | 1·35-1·40, p<0·0001 | 1·51 | 1·48-1·54, p<0·0001 |
| **Therapeutic anticoagulation** |  |  |  |  |
| No therapeutic anticoagulation | 1 | 1 | 1 | 1 |
| Therapeutic anticoagulation | 3·13 | 3·04-3·23, p<0·0001 | 1·13 | 1·09-1·17, p<0·0001 |
| **Hospital volume** |  |  |  |  |
| Low volume hospitals | 1 | 1 | 1 | 1 |
| Medium volume hospitals | 0·99 | 0·98-1·00, p<0·0001 | 1·01 | 0·96-1·06, p=0·818 |
| High volume hospitals | 0·92 | 0·91-0·93, p<0·0001 | 0·94 | 0·89-1·01, p=0·076 |

**Supp. Table 6: Risk factors of delayed surgery: univariable Odd’s Ratios and multivariable logistic regression model.** Delayed surgery denotes time from admission to surgery of longer than 12 hours in complicated appendicitis or longer than 24 hours in uncomplicated appendicitis. *Comorbidity score first introduced by Stausberg and colleagues^27^, whose validity has been affirmed in the German variant of the ICD-system. Known previous surgery was determined on coding of ICD side diagnoses: Previous organ loss by surgery: ICD Z90.3, Z90.4, Z90.5 or Z90.6 or Ostomy by previous abdominal surgery: ICD Z93.1, Z93.2, Z93.3, Z93.4, Z93.5 or Z93.6. Obesity is determined by side diagnosis ICD E66. Therapeutic anticoagulation was identified by side diagnosis coding of atrial fibrillation (ICD I48) or mechanic aortic valve replacement (ICD Z95.2); venous thrombosis or pulmonary artery embolism was not included since diagnosis coding, in contrast ot OPS coding, is not available with timing of the diagnosis, i.e. bias would be introduced in case venous thrombosis/thromboembolism occurred during the admission. For all patient records, a constant hospital identifier is provided, allowing for assignment to hospital tertiles treating an equal number of cases of acute appendicitis in the time window of this study (one third). Tertiles were identified on the basis of the caseload of each hospital, resulting in a hospital volume parameter of each patient records. Logit estimate 0·36, 95% Confidence-Interval (95 % CI) 0·34-0·37, area under the Receiving Operator Curve: 0·98, 95 % CI 0·97-0·99.

**Supp. Figure 3: Resulting recommendations by initial time of presentation.** Recommendations are based on the composite clinical endpoint and are weighed by Odd’s Ratios. Details in Table 3 and Supp. Table 4.
